# Effect of demographic features on morphometric variables of the knee joint: Sample of a 20 to 40-year-old Turkish population

**DOI:** 10.1097/MD.0000000000033253

**Published:** 2023-03-17

**Authors:** Muhammet Zeki Gültekin, Zeynep Keskin, Yaşar Mahsut Dinçel, Tuğba Arslan

**Affiliations:** a Department of Orthopedics and Traumatology, Konya City Hospital, Konya, Turkey; b Department of Radiology, Konya City Hospital, Konya, Turkey; c Department of Orthopedics and Traumatology, Faculty of Medicine, Tekirdag Namik Kemal University, Tekirdag, Turkey; d Department of Occupational Therapy, Faculty of Health Sciences, Karatekin University, Çankiri, Turkey.

**Keywords:** ACL angle, anterior cruciate ligament, BMI, Insall–Salvati index, knee joint, MRI, notch width index

## Abstract

This study aimed to investigate the relationship between body mass index (BMI), age, and sex and morphological risk factors that may cause internal knee injuries. The magnetic resonance images of 728 participants who met the inclusion criteria and had a mean age of 34.4 ± 6.8 years were analyzed retrospectively. Demographic differences were analyzed by measuring 17 morphological parameters known to be associated with internal knee injuries. Men had a higher anterior cruciate ligament length (ACLL), anterior cruciate ligament width, (ACLW) lateral femoral condylar width (LFCW), medial femoral condylar width (MFCW), lateral femoral condylar depth (LFCD), distal femoral width (DFW), and intercondylar femoral width (IFW) than women (*P < *.05). By contrast, the medial meniscus bone angle (MMBA) was lower in men than in women (*P < *.05). Women aged 31 to 40 years had a lower Insall–Salvati index (ISI) and lateral tibial posterior slope (LTPS) than those aged 21 to 30 years (*P < *.05), whereas men aged 31 to 40 years had a lower ISI than those aged 21 to 30 years (*P < *.05). Women with BMI ≥ 30 had a higher LFCW and MFCW but a lower ISI than those with BMI < 30 (*P < *.05). Men with BMI ≥ 30 had a higher LFCW, MFCW, DFW, and MMBA than those with BMI < 30 (*P < *.05). The use of value ranges structured according to demographic characteristics, rather than a single value range for all patient groups, may contribute to the evaluation and treatment of the morphological features that are thought to be effective in the development of internal knee injuries. These values may also shed light on future radiological risk scoring systems and artificial intelligence applications in medicine.

## 1. Introduction

Internal knee injuries are most common in young athletes^[1]^ with anterior cruciate ligament (ACL) injuries having one of the highest costs of treatment and follow-up^[2]^ (Atik, 2015). Only 50% of athletes who have undergone reconstruction for ACL and related intra-articular injuries can return to their former level of sports activity.^[3]^ In addition, knee injuries related to sports activities increase the incidence of osteoarthritis.^[4]^ These beneficial studies have suggested that preventive measures should be focused on before the development of ACL injuries.

Morphological features are thought to be risk factors for internal knee injuries. In this context, morphological features such as notch width, medial condylar width, lateral condylar width, bicondylar width, notch width index (NWI), notch width angle, medial tibial slope, lateral tibial slope, lateral posterior tibial slope, coronal tibial slope, depth of the medial tibial condyle, middle cartilage slope, medial tibial depth, lateral tibial meniscus bone angle, and lateral tibial meniscus bone angle have been investigated previously.^[5]^ However, a significant relationship between the risks of ACL injury, which is one of the most frequent knee injuries, and the morphological parameters of the knee has not been yet established.^[6]^ Thus, the reliability of these parameters, which are thought to be effective in internal knee injuries, considering demographic data, has become a matter of concern. Some studies have shown that some variables that may be morphological risk factors in injuries of the internal knee structures may be affected by demographic characteristics.^[7,8]^ For example, the width of the intercondylar notch has been reported to differ according to sex.^[7]^ A study also reported that the length and cross-sectional area of the ACL can change with age.^[8]^ Moreover, demographic studies usually appear to have focused on the relationship between sex and the intercondylar notch, and between age and the ACL size.^[9]^ However, to the best of our knowledge, no study has shown the effect of body mass index (BMI) on morphological variables. As such, the effects of demographics, including age, BMI, and sex on other morphological variables of the knee should be examined comprehensively.^[10]^

Given the aforementioned reasons, this study aimed to evaluate 17 morphological variables (i.e., anterior cruciate ligament length [ACLL], anterior cruciate ligament width [ACLW], anterior cruciate ligament inclination angle [ACLIA], Insall–Salvati index [ISI], Blumensaat angle, medial tibial plateau slope [MTPS], lateral tibial plateau slope [LTPS], lateral femoral condylar width [LFCW], anterior tibial translation [ATT], medial femoral condylar width [MFCW], medial femoral condylar depth [MFCD], lateral femoral condylar depth [LFCD], NWI, distal femoral width [DFW], intercondylar femoral width [IFW], medial meniscus bone angle [MMBA], and lateral meniscus bone angle [LMBA]), which are thought to increase the susceptibility to knee injuries, in the light of the literature, and to determine whether they changed with age, sex, or BMI.

## 2. Patients and Methods

The ethics approval for the study was obtained from the Non-Pharmaceutical and Non-Medical Device Research Ethics Committee of Necmettin Erbakan University (date: Feb 04, 2022; approval number: 2022/3641). The sample size was calculated with G*Power software using the LTPS data provided for female and male in the study by Han et al^[11]^ Accordingly, at least a total of 274 participants, 141 female and 133 male, had to be included in the study (effect size, 0.40; alpha = 0.05; 1-beta = 0.95; actual power, 100). For this reason, the data of 728 patients who underwent knee magnetic resonance imaging (MRI) between January 2020 and January 2022 in the radiology department of our hospital were analyzed. A total of 500 knee MRI examinations that met the study inclusion criteria were included in the study. Healthy knee MRI examinations were selected to focus on the effects of demographic characteristics on morphometric variables. Therefore, the inclusion criteria of the study were patients aged 20 to 40 years and who were Turkish. Patients with knee surgery, rheumatological diseases, knee infection, knee ligament injuries, fractures involving the knee, osteoarthritis, and neuromuscular diseases were excluded from the study. Informed consent was not obtained from the participants because of the retrospective design of the study and the anonymous analysis of the data.

**Figure 1.**
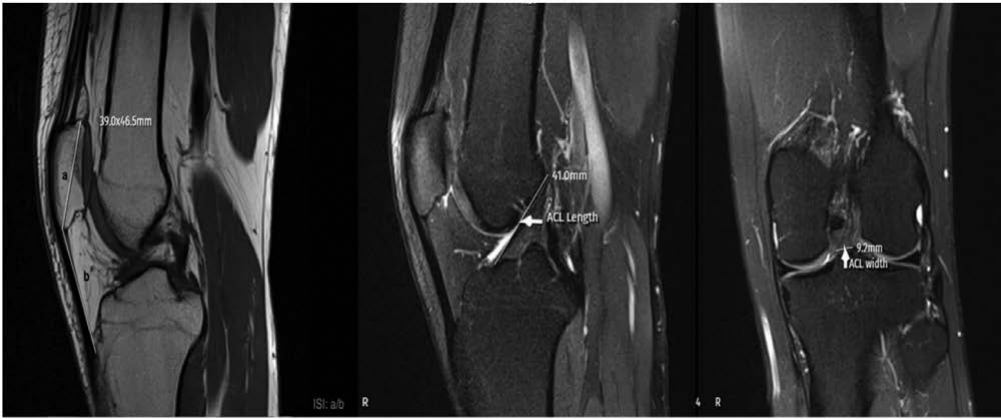
MRI measurement techniques of ISI, ACLL, and ACLW. ACLL = anterior cruciate ligament length, ACLW = anterior cruciate ligament width, ISI = Insall–Salvati index, MRI = magnetic resonance imaging.

**Figure 2.**
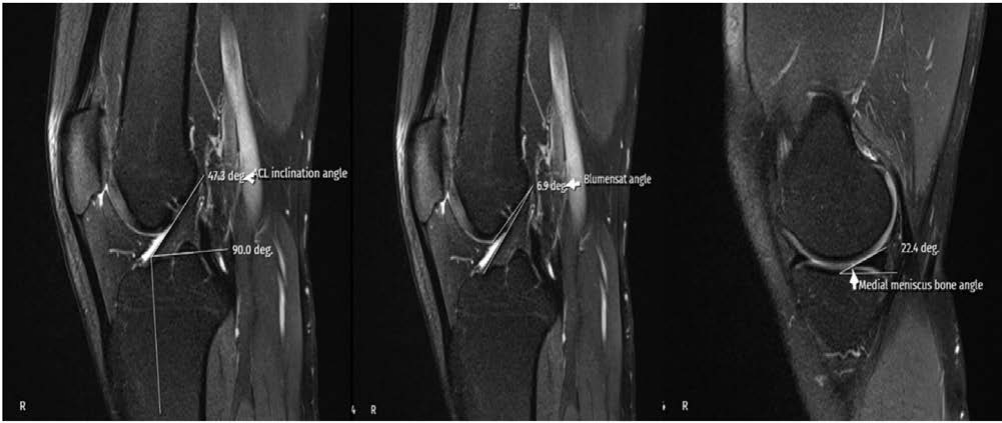
MRI measurement techniques of ACLIA, BA, and MMBA. ACLIA = anterior cruciate ligament inclination angle, BA = Blumensaat angle, MMBA = medial meniscus bone angle, MRI = magnetic resonance imaging.

**Figure 3.**
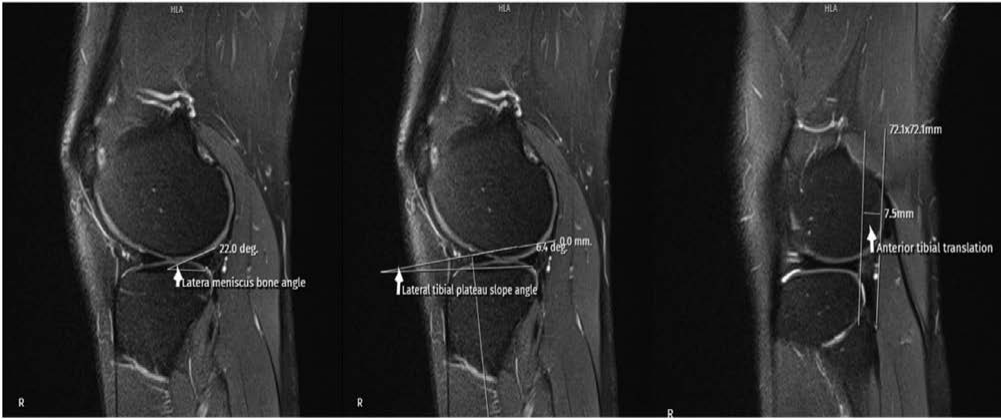
MRI measurement techniques of LMBA, LTPS, and ATT. ATT, anterior tibial translation; LMBA, lateral meniscus bone angle; LTPS, lateral tibial plateau slope; MRI, magnetic resonance imaging.

**Figure 4.**
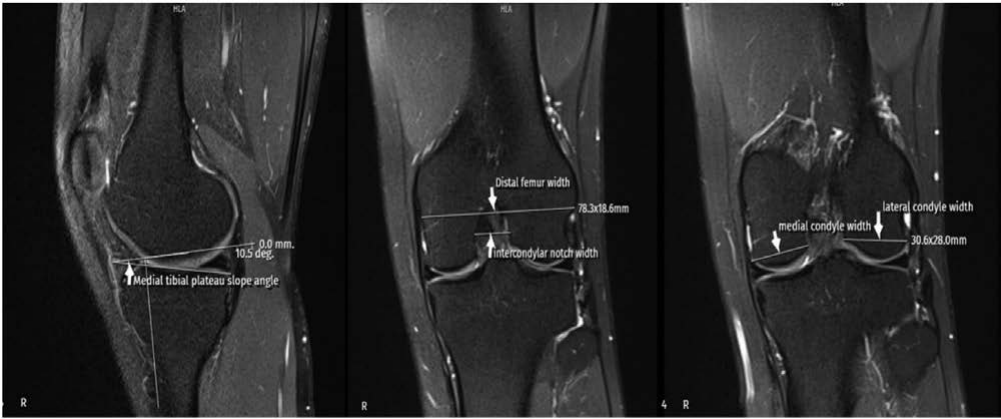
MRI measurement techniques of MTPS, IFW, MFCW, and LFCW. IFW = intercondylar femoral width, LFCW = lateral femoral condylar width, MFCW = medial femoral condylar width, MRI = magnetic resonance imaging, MTPS = medial tibial plateau slope.

**Figure 5.**
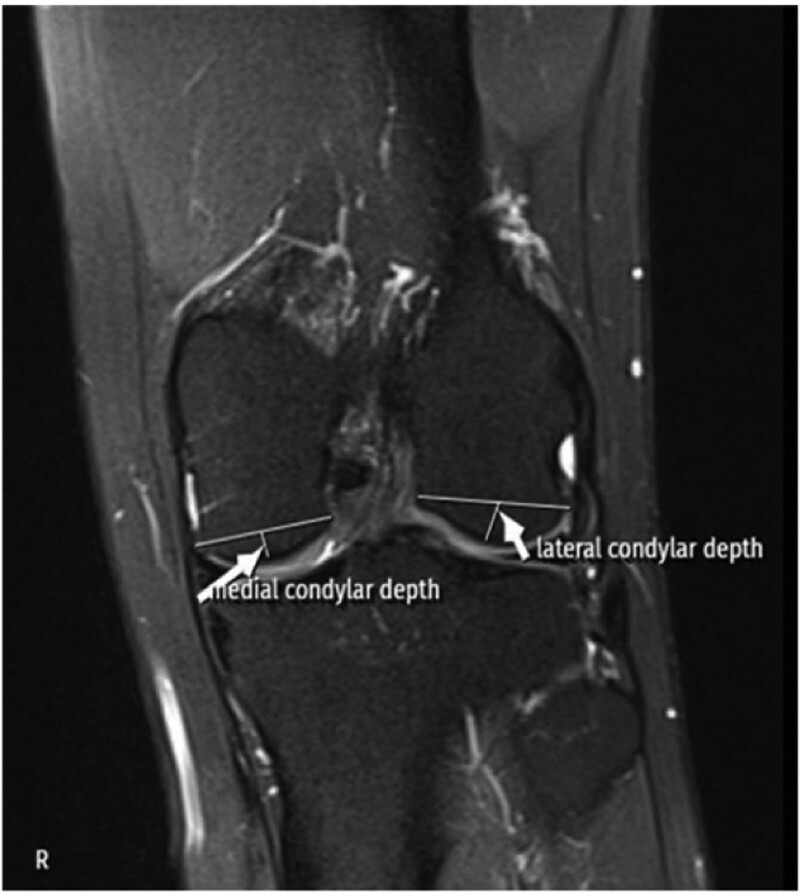
MRI measurement techniques of MFCD and LFCD. LFCD = lateral femoral condylar depth, MFCD = medial femoral condylar depth, MRI = magnetic resonance imaging.

An orthopedic surgeon with >10 years of experience in sports surgery and an experienced musculoskeletal radiologist examined the MRIs and measured the 17 morphological parameters using standard techniques previously described in the literature.^[8,9,12–18]^ The doctors who performed the measurements were blinded to the patient records. All MRIs were taken with a 1.5-T scanner (MAGNETOM Symphony; Siemens AG, Erlangen, Germany) with a 3-mm section thickness. Individual radiological measurements were performed virtually using the INFINITT PACS System (INFINITT Healthcare Co., Seoul, South Korea) with an accuracy of 0.1 mm for linear measurements and 0.1° for angular measurements. In all patients, the following values were measured using standard measurement techniques provided in the literature: BMI,^[9]^ ACLL,^[12]^ ACLW,^[8]^ ACLIA,^[12]^ ISI,^[13]^ BA,^[14]^ ATT,^[15]^ MTPS,^[16]^ LTPS,^[16]^ LFCW,^[17]^ MFCW,^[17]^ MFCD,^[17]^ LFCD,^[17]^ NWI,^[17]^ DFW,^[17]^ IFW,^[17]^ MMBA,^[18]^ and LMBA^[18]^ (Figs. 1–5).

The ACLL was determined in the sagittal section by measuring the distance between the center of the anterior cruciate ligament (ACL) attachment to the tibia and the center of the ACL attachment to the femur.^[12]^ The ACLW in the coronal section was determined by measuring the cross-sectional area made from the distal third of the ligament.^[8]^ In the sagittal section, the angle between the orthogonal line of the longitudinal axis of the tibia and the line formed between the femur and tibial attachment of the ligament was measured and recorded as the ACLIA.^[12]^ The ISI was defined as the ratio of the patellar tendon length to the maximum patella length.^[13]^ In the sagittal section where the entire Blumensaat line was noted, the angle formed by the Blumensaat line and the longitudinal axis of the femur was measured and defined as the body surface area.^[14]^ The ATT was measured in the sagittal section. Two vertical lines were drawn, one at the level of the posterior contour of the lateral tibial condyle and the other at the posterior part of the lateral femoral condyle, and the distance between these 2 lines was measured and noted as the ATT.^[15]^ The medial tibial plateau slope (MTPS) and LTPS were measured in the central sagittal section. The MTPS was defined as the angle between the orthogonal line of the tibial longitudinal axis and the line connecting the apex of the anterior and posterior cortex edges of the medial plateau of the tibia. The LTPS was defined as the angle between the orthogonal line of the tibial longitudinal axis and the line connecting the vertices of the anterior and posterior cortex edges of the tibial lateral plateau.^[16]^ The distances between the most medial and most lateral of the medial and lateral condyles in the axial section were recorded as the MFCW and LFCW. The distances between the most anterior and most posterior points of the medial and lateral condyles in the sagittal section were recorded as the MFCD and LFCD. A line was drawn through the popliteal grove in the central coronal section. The length of this line between the medial and lateral walls of the popliteal grove was measured and defined as the NW. The part of the line connecting the medial and lateral condyles was measured and recorded as the IFW. The NWI was calculated by dividing the NW by the IFW. The DFW was determined by measuring the distance from the lateral border of the lateral femoral condyle articular surface to the medial border of the medial femoral condyle articular surface in the axial section.^[17]^ The MMBA and LMBA were measured in the central sagittal section. For the MMBA, the line passing from the upper surface of the medial meniscus and the subchondral surface of the medial tibial plateau was drawn, and the angle between the 2 lines was measured. For the LMBA, the line passing from the upper surface of the lateral meniscus and the subchondral surface of the lateral tibial plateau was drawn, and the angle between the 2 lines was measured.^[18]^

The relationship between the abovementioned morphological measurements and demographic data such as age, sex, and BMI was statistically analyzed. The patients were divided into 2 groups based on age (those who were in their third or fourth decade of life), sex (male or female), and obesity (those who had a BMI of < 30 or ≥ 30 kg/m^2^). Descriptive statistics were expressed as mean, standard deviation, median, minimum, maximum, frequency, and percentage. The normality of the variables was checked using the Kolmogorov–Smirnov test. The Mann–Whitney *U* test was used to compare the quantitative data and the chi-square for the qualitative data. The IBM SPSS Statistics for Windows version 28.0 (IBM Corp., Armonk, NU) was used in all statistical analyses. The level of statistical significance was set at *P < *.05.

## 3. Results

The mean age of the patients was 34.4 ± 6.8 years (Table 1). Moreover, 141 (28.2%) patients were < 30 years old and 308 (61.6%) were female. About one-fifth of the participants had a BMI ≥ 30 (n = 107, 21.4%). The morphological variables of the patients are given in Table 2.

**Table 1:** Morphological and demographic data of the patients.

	Min-Max	Median	Mean ± SD/n(%)
Age, yr	18–40	37	34.4 ± 6.8
Age range, yr			
21–30			141 (28.2)
31–40			359 (71.8)
Gender			
Female			308 (61.6)
Male			192 (38.4)
Side			
Right			271 (54.2)
Left			229 (45.8)
Height, cm	130.0–204.0	166.0	167.6 ± 9.5
Weight, kg	43.0–135.0	76.0	76.5 ± 14.0
BMI	16.9–49.4	26.8	27.2 ± 4.5
BMI			
<30			393 (78.6)
≥30			107 (21.4)
ACLL	29.0–45.0	36.0	36.4 ± 2.9
ACLW	6.0–14.0	8.0	8.5 ± 1.4
ACLIA	39.0–56.0	48.0	47.6 ± 3.2
ISI	0.0–1.5	1.0	1.0 ± 0.1
BA	2.0–14.0	7.0	6.9 ± 2.2
ATT	0.0–6.0	2.0	1.7 ± 1.4
MTPS	2.0–19.0	8.0	8.6 ± 3.0
LTPS	2.0–16.0	7.0	7.1 ± 2.6
LFCW	23.0–39.0	30.0	30.2 ± 2.6
MFCW	21.0–34.0	26.0	26.5 ± 2.3
MFCD	2.0–8.0	5.0	5.2 ± 1.0
LFCD	3.0–9.0	5.0	5.0 ± 1.0
NWI	3.0–5.3	4.0	3.9 ± 0.4
DFW	62.0–93.0	76.0	76.7 ± 6.0
IFW	12.0–29.0	20.0	19.6 ± 2.5
MMBA	16.0–35.0	25.0	24.7 ± 3.0
LMBA	15.0–38.0	26.0	25.6 ± 3.8

ACLIA = anterior cruciate ligament inclination angle, ACLL = anterior cruciate ligament length, ACLW = anterior cruciate ligament width, and LMBA = lateral meniscus bone angle, ATT = anterior tibial translation, BA = Blumensaat angle, DFW = distal femoral width, IFW = intercondylar femoral width, ISI = Insall-Salvati index, LFCD = lateral femoral condylar depth, LFCW = lateral femoral condylar width, LTPS = lateral tibial plateau slope, MFCD = medial femoral condylar depth, MFCW = medial femoral condylar width, MMBA = medial meniscus bone angle, MTPS = medial tibial plateau slope, NWI = notch width index.

**Table 2:** Results of the morphological measurements by gender.

	Female			Male			*P* *
	Mean ± SD	Median	Min-Max	Mean ± SD	Median	Min-Max	
ACLL	35.34 ± 2.43	35.0	29.0–43.0	38.22 ± 2.76	38.0	32.0–45.0	**.000**
ACLW	8.03 ± 1.21	8.0	6.0–13.0	9.20 ± 1.41	9.0	6.0–14.0	**.000**
ACLIA	47.64 ± 3.18	48.0	39.0–56.0	47.63 ± 3.24	47.5	39.0–56.0	.808
ISI	1.01 ± 0.13	1.0	0.7–1.5	0.99 ± 0.15	1.0	0.0–1.4	.225
BA	6.98 ± 2.24	7.0	2.0–14.0	6.76 ± 2.21	7.0	2.0–13.0	.380
ATT	1.73 ± 1.46	1.0	0.0–6.0	1.76 ± 1.42	2.0	0.0–6.0	.688
MTPS	8.72 ± 2.95	8.0	2.0–17.0	8.47 ± 2.99	8.0	2.5–19.0	.378
LTPS	7.22 ± 2.68	7.0	2.0–16.0	6.83 ± 2.50	7.0	2.0–14.0	.098
LFCW	28.97 ± 2.00	29.0	23.0–34.0	32.2 ± 2.26	32.0	24.0–39.0	**.000**
MFCW	25.75 ± 1.94	26.0	21.0–34.0	27.77 ± 2.31	28.0	21.0–33.0	**.000**
MFCD	5.06 ± 0.88	5.0	2.0–8.0	5.40 ± 1.04	5.0	3.0–8.0	**.001**
LFCD	4.81 ± 0.90	5.0	3.0–8.0	5.41 ± 1.10	5.0	3.0–9.0	**.000**
NWI	3.94 ± 0.39	3.9	3.0–5.2	3.95 ± 0.37	4.0	3.1–5.3	.492
DFW	73.40 ± 4.00	73.0	62.0–86.0	82.07 ± 4.65	82.0	68.0–93.0	**.000**
IFW	18.69 ± 2.10	19.0	12.0–27.0	20.99 ± 2.45	21.0	15.0–29.0	**.000**
MMBA	24.93 ± 3.11	25.0	16.0–35.0	24.36 ± 2.77	24.0	18.0–31.0	**.030**
LMBA	25.65 ± 3.81	26.0	15.0–37.0	25.63 ± 3.87	25.5	18.0–38.0	.813

Significant *P* values are written in bold.

ACLIA = anterior cruciate ligament inclination angle, ACLL = anterior cruciate ligament length, ACLW = anterior cruciate ligament width, and LMBA = lateral meniscus bone angle, ATT = anterior tibial translation, BA = Blumensaat angle, DFW = distal femoral width, IFW = intercondylar femoral width, ISI = Insall-Salvati index, LFCD = lateral femoral condylar depth, LFCW = lateral femoral condylar width, LTPS = lateral tibial plateau slope, MFCD = medial femoral condylar depth, MFCW = medial femoral condylar width, MMBA = medial meniscus bone angle, MTPS = medial tibial plateau slope, NWI = notch width index.

* Mann–Whitney *U* test.

Men had a higher ACLL, ACLW, LFCW, MFCW, LFCD, DFW, and IFW than women (*P < *.05). On the contrary, MMBA was lower in men than in women (*P < *.05). The LTPS in men aged 21 to 30 years was lower than that of women aged 21 to 30 years (*P < *.05). The ACLIA, ISI, BA, ATT, MTPS, MFCD, NWI, MMBA, and LMBA in patients aged 21 to 30 years did not show a significant difference between men and women, whereas the ACLIA, ISI, BA, ATT, MTPS, LTPS, MFCD, NWI, and LMBA were comparable in men and women aged 31 to 40 years.

Women aged 31 to 40 years had a lower ISI and LTPS than those aged 21 to 30 years (*P < *.05), whereas the ACLL, ACLW, ACLIA, BA, ATT, MTPS, LFCW, MFCW, MFCD, LFCD, NWI, DFW, IFW, MMBA, and LMBA were not significantly different between the 2 age groups (Table 3).

**Table 3:** Comparison of the morphological variables in men and women according to age.

	Female					Male				
	21–30 yr		31–40 yr		*P* *	21–30 yr		31–40 yr		*P* *
	Mean ± SD	Median	Mean ± SD	Median		Mean ± SD	Median	Mean ± SD	Median	
ACLL	35.3 ± 2.4	35.0	35.3 ± 2.4	35.0	.673	38.7 ± 2.9	39.0	38.0 ± 2.7	38.0	.128
ACLW	8.1 ± 1.4	8.0	8.0 ± 1.2	8.0	.717	9.4 ± 1.6	9.0	9.1 ± 1.3	9.0	.326
ACLIA	47.5 ± 3.1	48.0	47.7 ± 3.2	48.0	.702	47.7 ± 3.2	47.0	47.6 ± 3.3	48.0	.784
ISI	1.04 ± 0.13	1.00	1.00 ± 0.13	1.00	**.035**	1.03 ± 0.16	1.00	0.98 ± 0.14	1.00	**.038**
BA	7.3 ± 2.3	7.0	6.92 ± 2.2	7.0	.147	6.7 ± 2.3	6.0	6.8 ± 2.2	7.0	.515
ATT	1.9 ± 1.5	2.0	1.7 ± 1.5	1.0	.178	1.8 ± 1.5	2.0	1.7 ± 1.4	2.0	.773
MTPS	8.9 ± 3.1	9.0	8.7 ± 2.9	8.0	.559	8.1 ± 2.9	8.0	8.7 ± 3	8.0	.217
LTPS	8.0 ± 2.9	8.0	7.0 ± 2.6	6.0	**.003**	6.6 ± 2.6	6.0	7.0 ± 2.5	7.0	.258
LFCW	28.7 ± 2	29.0	29.1 ± 2	29.0	.147	32.5 ± 2.2	33.0	32.1 ± 2.3	32.0	.194
MFCW	25.5 ± 1.7	25.0	25.8 ± 2	26.0	.175	27.6 ± 2.3	27.0	27.9 ± 2.3	28.0	.287
MFCD	5.1 ± 0.9	5.0	5.0 ± 0.9	5.0	.599	5.6 ± 1.2	5.0	5.3 ± 0.9	5.0	.418
LFCD	4.9 ± 0.9	5.0	4.8 ± 0.9	5.0	.355	5.6 ± 1.2	6.0	5.3 ± 1	5.0	.115
NWI	3.9 ± 0.4	3.9	3.9 ± 0.4	3.9	.512	3.9 ± 0.3	4.0	4.0 ± 0.4	4.0	.985
DFW	73.1 ± 4.2	72.0	73.5 ± 4	74.0	.257	82.2 ± 4	82.0	82.0 ± 5	82.0	.778
IFW	18.7 ± 2.1	19.0	18.7 ± 2.1	19.0	.659	20.9 ± 1.8	21.0	21.0 ± 2.7	21.0	.836
MMBA	24.5 ± 3	25.0	25.1 ± 3.1	25.0	.154	24.2 ± 2.8	25.0	24.4 ± 2.8	24.0	.758
LMBA	25.6 ± 3.5	26.0	25.7 ± 3.9	25.0	.875	25.2 ± 3.5	25.0	25.9 ± 4	26.0	.374

Significant *P* values are written in bold.

ACLIA = anterior cruciate ligament inclination angle, ACLL = anterior cruciate ligament length, ACLW = anterior cruciate ligament width, and LMBA = lateral meniscus bone angle, ATT = anterior tibial translation, BA = Blumensaat angle, DFW = distal femoral width, IFW = intercondylar femoral width, ISI = Insall-Salvati index, LFCD = lateral femoral condylar depth, LFCW = lateral femoral condylar width, LTPS = lateral tibial plateau slope, MFCD = medial femoral condylar depth, MFCW = medial femoral condylar width, MMBA = medial meniscus bone angle, MTPS = medial tibial plateau slope, NWI = notch width index.

* Mann–Whitney *U* test.

Men aged 31 to 40 years had a lower ISI than those aged 21 to 30 years (*P < *.05), whereas the ACLL, ACLW, ACLIA, BA, ATT, MTPS, LTPS, LFCW, MFCW, MFCD, LFCD, NWI, DFW, IFW, MMBA, and LMBA were not significantly different between the 2 age groups (Table 3).

Women with BMI ≥ 30 had higher LFCW and MFCW but lower ISI than those with BMI < 30 (*P < *.05). The ACLL, ACLW, ACLIA, BA, ATT, MTPS, LTPS, MFCD, NWI, DFW, IFW, MMBA, and LMBA were not significantly different between the 2 BMI groups (Table 4).

**Table 4:** Comparison of the morphological variables in men and women according to BMI.

	Female					Male				
	BMI < 30		BMI ≥ 30		*P* *	BMI < 30		BMI ≥ 30		*P* *
	Mean ± SD	Median	Mean ± SD	Median		Mean ± SD	Median	Mean ± SD	Median	
ACLL	35.3 ± 2.5	35.0	35.5 ± 2.2	35.0	.422	38.1 ± 2.8	38.0	38.8 ± 2.5	39.0	.311
ACLW	8.1 ± 1.2	8.0	8.0 ± 1.2	8.0	.642	9.2 ± 1.4	9.0	9.1 ± 1.5	9.0	.779
ACLIA	47.8 ± 3.2	48.0	47.3 ± 3.2	47.0	.290	47.7 ± 3.3	47.5	47.2 ± 2.6	47.5	.558
ISI	1.02 ± 0.13	1.00	0.98 ± 0.13	1.00	**.016**	1.0 ± 0.2	1.0	1.0 ± 0.1	1.0	.910
BA	7.0 ± 2.2	7.0	6.9 ± 2.3	7.0	.806	6.8 ± 2.2	7.0	6.7 ± 2.0	6.0	.871
ATT	1.7 ± 1.4	1.0	1.8 ± 1.6	2.0	.827	1.7 ± 1.4	2.0	1.9 ± 1.3	2.0	.449
MTPS	8.7 ± 2.9	8.0	8.8 ± 3.1	8.0	.702	8.4 ± 2.8	8.0	9.2 ± 4.1	8.0	.704
LTPS	7.3 ± 2.8	7.0	7.0 ± 2.3	7.0	.594	6.7 ± 2.4	7.0	7.5 ± 2.8	7.0	.252
LFCW	28.8 ± 2.1	29.0	29.3 ± 1.7	30.0	**.023**	32.1 ± 2.2	32.0	33.3 ± 2.4	33.5	**.010**
MFCW	25.6 ± 2.0	25.0	26.1 ± 1.7	26.0	**.039**	27.6 ± 2.3	27.5	28.8 ± 2.1	29.5	**.010**
MFCD	5.1 ± 0.9	5.0	5.0 ± 0.8	5.0	.737	5.4 ± 1.1	5.0	5.1 ± 0.8	5.0	.125
LFCD	4.8 ± 0.9	5.0	4.8 ± 0.9	5.0	.604	5.4 ± 1.1	5.3	5.2 ± 1.1	5.0	.260
NWI	3.9 ± 0.4	3.9	4.0 ± 0.4	4.0	.252	4.0 ± 0.4	4.0	3.9 ± 0.3	4.0	.740
DFW	73.2 ± 4.1	73.0	74.0 ± 3.7	74.0	.076	81.8 ± 4.4	82.0	83.8 ± 5.8	85.0	**.044**
IFW	18.7 ± 2.1	19.0	18.7 ± 2.0	19.0	.992	21.0 ± 2.4	21.0	21.3 ± 2.6	20.5	.771
MMBA	24.9 ± 3.0	25.0	25.1 ± 3.4	25.0	.596	24.2 ± 2.8	24.0	25.5 ± 2.6	25.0	**.029**
LMBA	25.5 ± 3.9	25.0	26.0 ± 3.7	26.0	.260	25.7 ± 3.9	25.5	24.8 ± 4.0	25.5	.273

Significant *P* values are written in bold.

ACLIA = anterior cruciate ligament inclination angle, ACLL = anterior cruciate ligament length, ACLW = anterior cruciate ligament width, and LMBA = lateral meniscus bone angle, ATT = anterior tibial translation, BA = Blumensaat angle, DFW = distal femoral width, IFW = intercondylar femoral width, ISI = Insall-Salvati index, LFCD = lateral femoral condylar depth, LFCW = lateral femoral condylar width, LTPS = lateral tibial plateau slope, MFCD = medial femoral condylar depth, MFCW = medial femoral condylar width, MMBA = medial meniscus bone angle, MTPS = medial tibial plateau slope, NWI = notch width index.

* Mann–Whitney *U* test.

Men with BMI ≥ 30 had higher LFCW, MFCW, DFW, and MMBA than those with BMI < 30 (*P < *.05). The ACLL, ACLW, ACLIA, ISI, BA, ATT, MTPS, LTPS, MFCD, NWI, IFW, and LMBA did not exhibit any significant difference between the 2 BMI groups (Table 4).

## 4. Discussion

The NWI aims to standardize the notch width relative to the overall distal femur width. Similar to the results of our study, the NWI and ACL dimensions (ACLL and ACLW) in women were smaller than those in men.^[7]^ Although the narrowness of the intercondylar notch was found to be associated with an increased risk of non-contact ACL injury,^[19]^ studies have reported conflicting results in the examination of the significance of the differences in NWI between men and women with ACL injuries.^[5]^ Similar to our study, a previous study also suggested a parallel relationship between the notch size (IFW) and ACL diameter.^[20]^

Women have smaller ACL than men when standardized for body weight and height; however, this difference is not correlated with ACL injury.^[7,20]^ We found similar results; however, the correlation between ACL size and ACL injury was not the focus of the present study. Given the higher incidence of ACL injury in women, it should be considered multifactorial. In this regard, neuromuscular and proprioceptive training focusing on injury prevention is needed.

Studies that attempted to establish a relationship between anthropometric data and ACLL have reported inconsistent results.^[21,22]^ Brown et al confirmed a strong positive correlation between ACLL and patient height and suggested that patient height can be a predictor of appropriate graft length in ACL reconstruction.^[21]^ However, Denti et al found no significant relationship between the length of the intra-articular ACL graft and patient height and weight.^[22]^ In our study, the ACLL in men was significantly higher than that in women. However, an overall evaluation of all patients showed that ACLL was not affected by age, side, or BMI.

ACL thickness at the tibial insertion point (ACLW) was 8 mm in our study population, a value lower than that in the Western population,^[23]^ but slightly larger than that in others.^[24]^ The difference might be related to the measurement technique because we measured the ACLW at its tibial attachment, whereas Tan et al measured at the midpoint of the ACL.^[24]^ Considering the fan-like structure of ACL fibers, our measurement at the tibial insertion point was wider.

Since the anatomical placement of the graft in the tunnel is important in ACL reconstruction, researchers have investigated the ACLIA in different populations. Both Illingworth et al and Kupczik et al recounted that this value ranged between 43 and 57 degrees.^[25,26]^ Similar to these studies, our ACLIA measurements range between 39 and 56 degrees. In our study, the ACLIA was unaffected by sex, side, BMI, or age.

The ISI was another important parameter in our study because it has been associated with osteoarthritis, ligament injuries, and osteochondropathies, which are both material and moral burdens in sports and medicine.^[27]^ Studies showing that the ISI is associated with meniscal injury are limited.^[18]^ Thus, we investigated the demographic distribution of the frequently measured ISI. In our study, the ISI was higher in women with BMI ≥ 30. This fact should be considered when evaluating the ISI and the risk of internal knee injuries in women who have obesity.

The LTPS and LMBA are important geometric measurements of the knee joint. Therefore, they are of interest in ACL injury and reconstruction. Recent studies have shown that MTPS and LTPS are independent risk factors for primary and recurrent ACL damage.^[18,28]^ In addition, biomechanical and clinical evidence indicates that the lateral meniscus contributes significantly to the stability of the rotating knee. LMBA was also found to be an independent risk factor for ACL injuries.^[25]^ In our study, these independent risk factors were not affected by BMI; therefore, they can be safely measured.

This study had some limitations. First, patients aged 21 to 40 years were included in the study. Therefore, the results of the study cannot explain whether morphological differences in individuals aged < 21 years and > 40 years are affected by demographic characteristics. Second, the patients belonged to a single ethnicity. Thus, our results may be insufficient to reveal the facts about different ethnic origins. Third, morphometric variables were made once by a single researcher. Therefore, intraobserver and interobserver biases may not have been prevented. Considering all these limitations, we that studies with a wider patient population and more observers will better guide the researchers in this field.

## 5. Conclusion

The results of the study indicated that some morphological variables that may be associated with internal knee injuries are affected by age, sex, and BMI. The ISI and LTPS in women and only ISI in men differ according to age. The ACLL, ACLW, LFCW, MFCW, LFCD, DFW, and IFW values vary by sex. The LFCW, MFCW, DFW, and MMBA results in men, and the ISI, LFCW, and MFCW results in women were associated with BMI. The use of value ranges structured according to demographic characteristics, rather than a single value range for all patient groups, may contribute to the evaluation and treatment of the morphological features that are thought to be effective in the development of internal knee injuries. These values may also shed light on future radiological risk scoring systems and artificial intelligence applications in medicine.

## Author contributions

**Conceptualization:** Muhammet Zeki Gültekin, Yaşar Mahsut Dinçel, Tuğba Arslan.

**Data curation:** Muhammet Zeki Gültekin, Zeynep Keskin.

**Methodology:** Muhammet Zeki Gültekin, Zeynep Keskin.

**Supervision:** Yaşar Mahsut Dinçel.

**Writing – original draft:** Zeynep Keskin, Tuğba Arslan.

**Writing – review & editing:** Yaşar Mahsut Dinçel, Tuğba Arslan.
